# PASMVS: A perfectly accurate, synthetic, path-traced dataset featuring specular material properties for multi-view stereopsis training and reconstruction applications

**DOI:** 10.1016/j.dib.2020.106219

**Published:** 2020-08-24

**Authors:** André Broekman, Petrus Johannes Gräbe

**Affiliations:** Department of Civil Engineering, University of Pretoria, South Africa - University of Pretoria, Lynnwood Road, Hatfield, Pretoria 0002, South Africa

**Keywords:** Multi-view stereopsis, 3D reconstruction, Synthetic data, Ground truth depth map, Blender

## Abstract

A Perfectly Accurate, Synthetic dataset for Multi-View Stereopsis (PASMVS) is presented, consisting of 400 scenes and 18,000 model renderings together with ground truth depth maps, camera intrinsic and extrinsic parameters, and binary segmentation masks. Every scene is rendered from 45 different camera views in a circular pattern, using Blender's path-tracing rendering engine. Every scene is composed from a unique combination of two camera focal lengths, four 3D models of varying geometrical complexity, five high definition, high dynamic range (HDR) environmental textures to replicate photorealistic lighting conditions and ten materials. The material properties are primarily specular, with a selection of more diffuse materials for reference. The combination of highly specular and diffuse material properties increases the reconstruction ambiguity and complexity for MVS reconstruction algorithms and pipelines, and more recently, state-of-the-art architectures based on neural network implementations. PASMVS serves as an addition to the wide spectrum of available image datasets employed in computer vision research, improving the precision required for novel research applications.

## Specifications Table

**Table:** 

Subject	Computer Vision and Pattern Recognition
Specific subject area	Multi-view stereopsis and 3D reconstruction from images
Type of data	Image Depth maps CSV 3D model geometry
How data were acquired	A photorealistic virtual environment was created using Blender and rendered with the path-tracing rendering engine (Cycles). Different combinations of popular geometry models, surface materials, environmental textures and camera parameters were used to render the large variety of data samples. The binary segmentation maps were rendered alongside the colour images through assigning different material identification numbers for the geometry models and environmental textures. The ground truth depth map was also obtained during the same rendering pass by exporting the camera's Z-buffer (distance between the camera and intersecting geometry for every pixel of the imaging sensor). The intrinsic and extrinsic camera files were exported as a single (comma-separated value) CSV file for every scene.
Data format	Raw
Parameters for data collection	Using a constant, circular path for the camera around the centre point of the model, all possible combinations of model geometries, environmental lighting textures, model material properties and the camera focal lengths were rendered.
Description of data collection	Using Blender, path-traced images, ground truth depth map and binary segmentation maps were rendered using different models. 400 scenes in total were rendered using a combination of ten, primarily specular materials, five environmental textures, four models and two focal lengths. 45 views per scene yield a total of 18,000 synthetic samples. Intrinsic and extrinsic camera parameters were exported for each scene for generating camera matrices. Post-processing corrects Blender's rendered distance maps to depth maps.
Data source location	Institution: Department of Civil Engineering, University of Pretoria City: Pretoria Country: South Africa
Data accessibility	Repository name: Mendeley Data Data identification number: 10.17632/fhzfnwsnzf.2

## Value of the Data

• The data enables the development of accurate, sub-millimetre accurate reconstruction pipelines and architectures required for sensitive optical metrology applications, such as the geometry measurements of railway profiles.
• PASMVS can be used for benchmarking photogrammetric pipelines and training MVS neural network architectures [1] that are dependant on large, accurate ground truth datasets.
• The data structure and file formats are agnostic to most state-of-the-art, MVS neural network implementation requirements [1] such as BlendedMVS [2].
• Ablation-specific experiments can be performed by varying the illumination, geometry, material properties and camera focal length parameters in isolation.

## 1 Data Description

MVS reconstruction pipelines, particularly state-of-the-art developments that are based on neural network implementations, require both a large sample distribution of photorealistic image sequences alongside accurate ground truth depth maps, to learn and generalise effectively. Taking inspiration from recent synthetic data generation approaches [3], [4], [5], [6] and its application in MVS [7], [8], [9], [10], PASMVS [11] was developed to address some of the limitations presented by existing datasets. Existing methods typically integrate optical sensors to generate a digitized ground truth. For example, BlendedMVS [2] employs an inexpensive, unmanned aerial vehicle (UAV) to photograph relatively large urban areas and monuments that are reconstructed using traditional photogrammetry. For smaller dimensions, a laser scanner can be used to generate the ground truth [12] with a finite resolution (0.25 mm) and accuracy (0.05 mm). By contrast, PASMVS utilises a digital ground truth and processing pipeline that provides perfect accuracy (0 mm), independent of the scale, dimensions and instrumentation characteristics such as inherent noise and limited resolution. This digital approach is required for the development of datasets that are used for sub-millimetre accuracy reconstruction applications, such as the reconstruction of railway environments for the purpose of geometry measurements [13]. Additionally, the ground truth of highly specular material surfaces, for example steel, are difficult to reconstruct accurately using existing methods. The implementation of neural networks for MVS reconstruction pipelines [1], whilst accommodating these more challenging material characteristics, are limited in their reconstruction accuracy by the current selection of datasets available that these networks are currently trained on. PASMVS serves both as validation of a neural network's ability to encode the reconstruction process for specular materials in addition to a providing a ground truth with perfect accuracy for improved reconstruction accuracy.

**Fig. 1:**
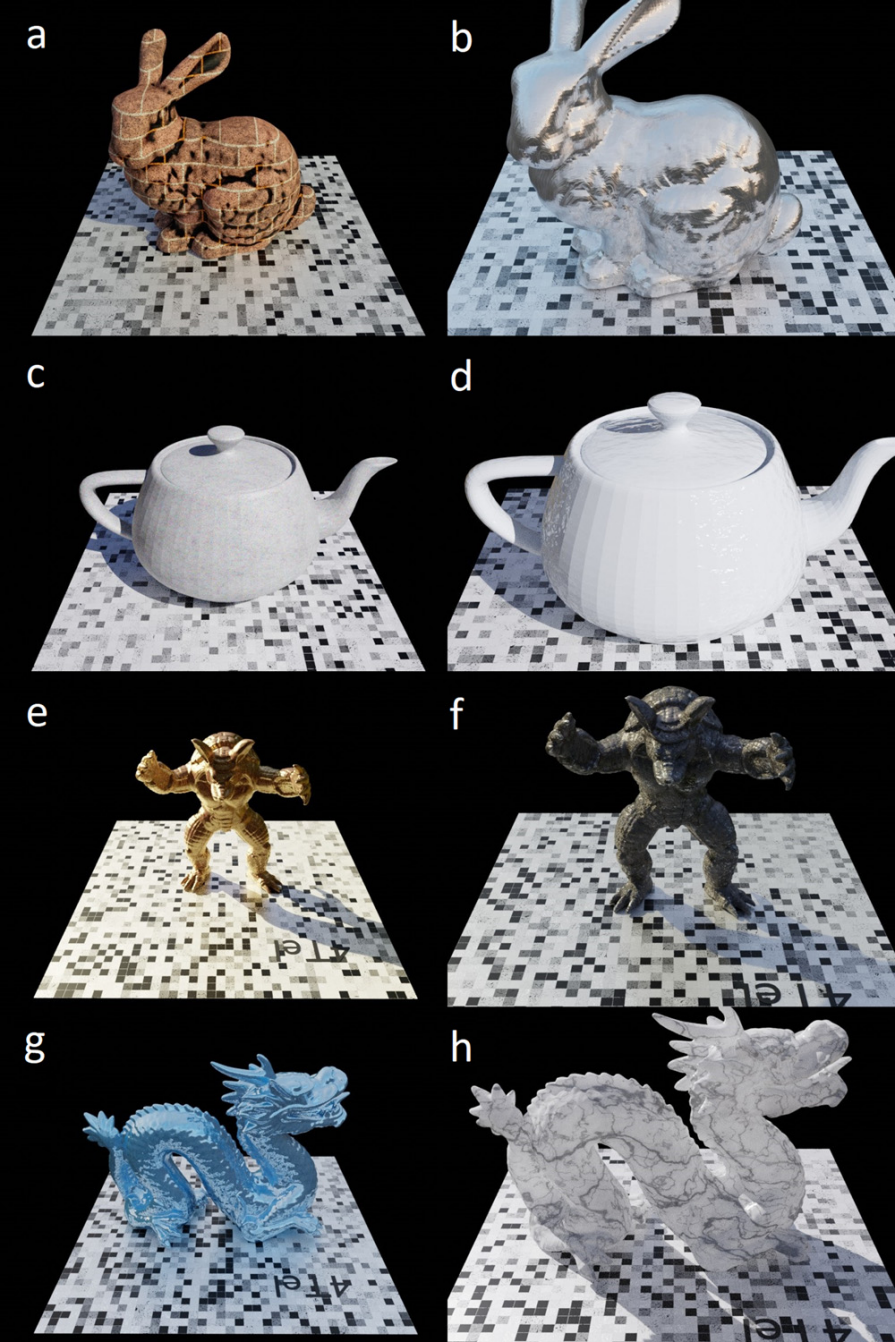
PASMVS samples illustrating the selection of models, variation in illumination, material properties and camera focal lengths. (a) bunny, bricks, 35 mm, (b) bunny, brushedmetal, 50 mm, (c) teapot, concrete, 35 mm, (d) teapot, ceramic, 50 mm, (e) armadillo, copper, 25 mm, (f) armadillo, grungemetal, 50 mm, (g) dragon, piano, 25 mm, (h) dragon, marble, 50 mm.

For the proposed dataset, the selected model is positioned above a square ground plane in the centre of the scene and sized to occupy most of the camera frame. A camera is rotated around the model in a circular path, generating a total of 45 frames per scene. Four models were selected; these models are the ubiquitous bunny, dragon and armadillo models developed by the Stanford Computer Graphics Laboratory [14], in addition to the Utah teapot [15]. These models are commonly used in numerical and computer vision applications. For every model, a unique combination of ten materials, five HDR environmental background textures and two camera focal lengths (35 mm and 50 mm) were used for the scenes. These unique combinations yield a total of 400 scenes and 45 camera views per scene, for a total of 18,000 samples for the PASMVS dataset. The “PASMVS.blend” Blender source file is available from the data repository [11]. Fig. 1 illustrates a sample of 8 scenes illustrating the variation of model selection, environmental illumination, material properties and camera focal length. Every scene is assigned a unique folder number, i.e. “armadillo10bricks35mm”, that corresponds to the concatenation of the selected model, environment texture identification number, descriptive texture name and the focal length of the camera. For ease of implementation with MVSNet [1] or similar neural network architectures, the scene folders were randomly selected and divided according to a 85%−15% train-validation split; a list of all scenes, training scenes and validation scenes are stored in the requisite “all_list.txt”, “training_list.txt” and “validation_list.txt” text files respectively. The “index.csv” CSV file provides a convenient reference to all 18,000 sample files, linking the corresponding files and relative data path.

The camera information file for every scene is exported as a CSV and stored in the scene folder as “scene.csv”. All signed float values are stored to a length of 5 decimal places. The following parameters are stored in the scene file:

• frame: frame number identification increasing from 0 through 44 for every camera view.
• posX, posY, posZ: position vector (measured in meters) of the camera's origin point in Blender's world coordinate system; signed float.
• rotX, rotY, rotZ: rotation vector (measured in degrees, XYZ) of the camera coordinate system; signed float.
• resX, rexY: resolution (in pixels) of the sensor image; unsigned integer.
• focalLength: focal length of the camera (measure in millimetres); unsigned integer.
• sensorWidth: width of the camera's imaging sensor (in millimetres); unsigned integer.

The ground plane consists of a randomised checkerboard pattern to add reference features during reconstruction. The following ten materials are implemented for the synthetic dataset:

• bricks: mottled brick texture with grouting pattern; low specularity.
• brushedmetal: uniformly brushed metal; very high specularity.
• ceramic: uniform, white ceramic, reminiscent of porcelain; high specularity.
• checkerboard: swirly checkerboard primary with contrasting primary colours. Low specularity.
• concrete: uniform, finely textured concrete; low specularity.
• copper: uniform, red-tinted copper; high specularity.
• grungemetal: bronze-coloured metal with non-uniform patches of rough metal texture; medium specularity.
• marble: uniform white marble contrasted with fine, black vein details.
• piano: blue-tinted piano ivory; high specularity.
• steel: stainless steel; very high specularity.

**Fig. 2:**
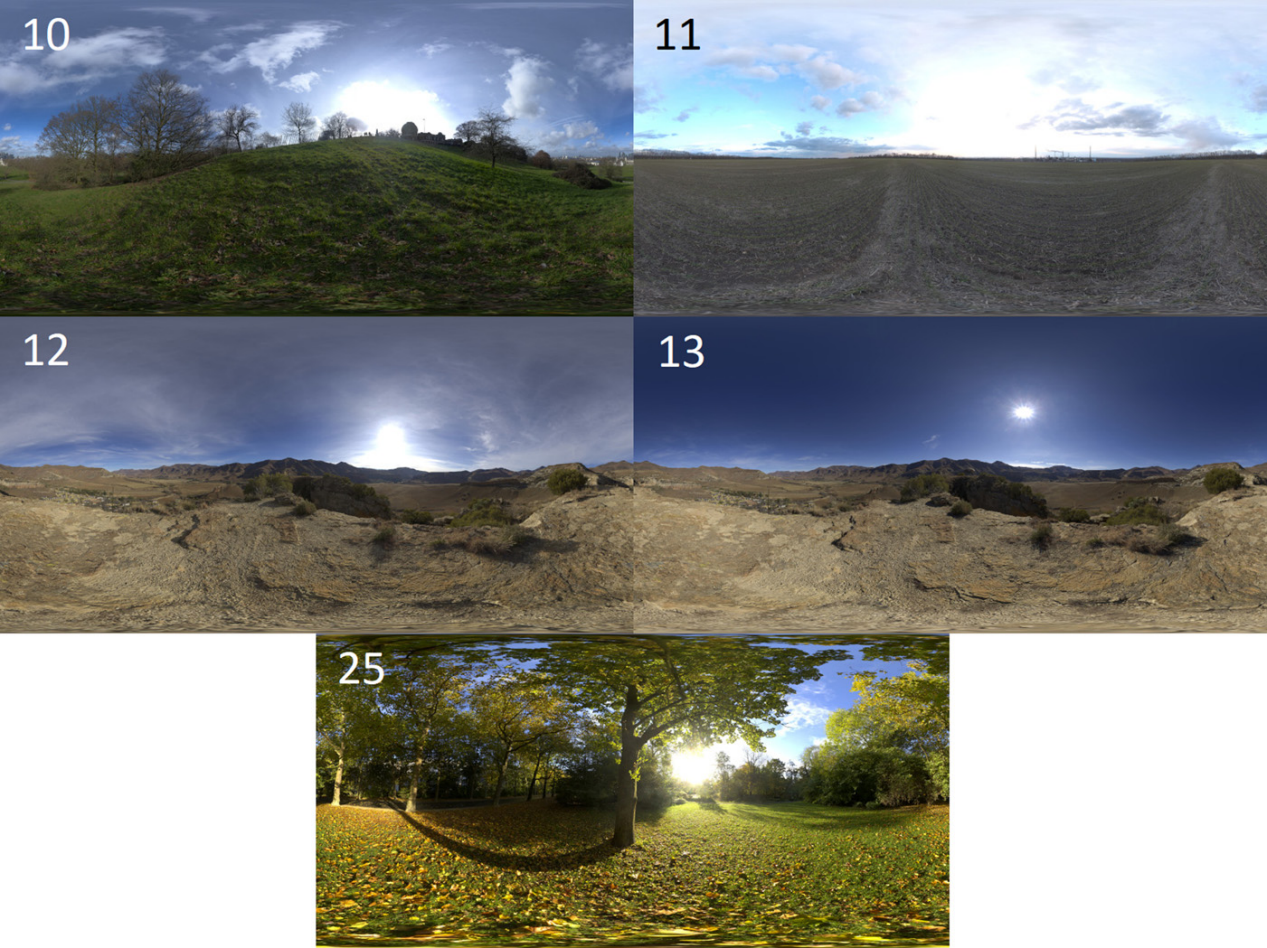
Illustration of the high definition environmental lighting textures used for photorealistic scene illumination. (10) greenwich_park_8, (11) industrial_sunset_8k, (12) kiara_3_morning_8k, (13) kiara_4_mid-morning_8k, (25) sunny_vondelpark_8k.

For the environmental lighting textures, five high-definition textures (8 K resolution) sourced from HDRIHaven [16] were implemented, replicating the illumination from a variety of natural environments. Fig. 2 illustrates the equirectangular projections of the maps along with their respective identification numbers used as part of the folder naming scheme, in addition to the original filename.

For every unique scene folder, the output files are subdivided and stored in four sub-folders, each described below.

## 2 blended_images

**Fig. 3:**
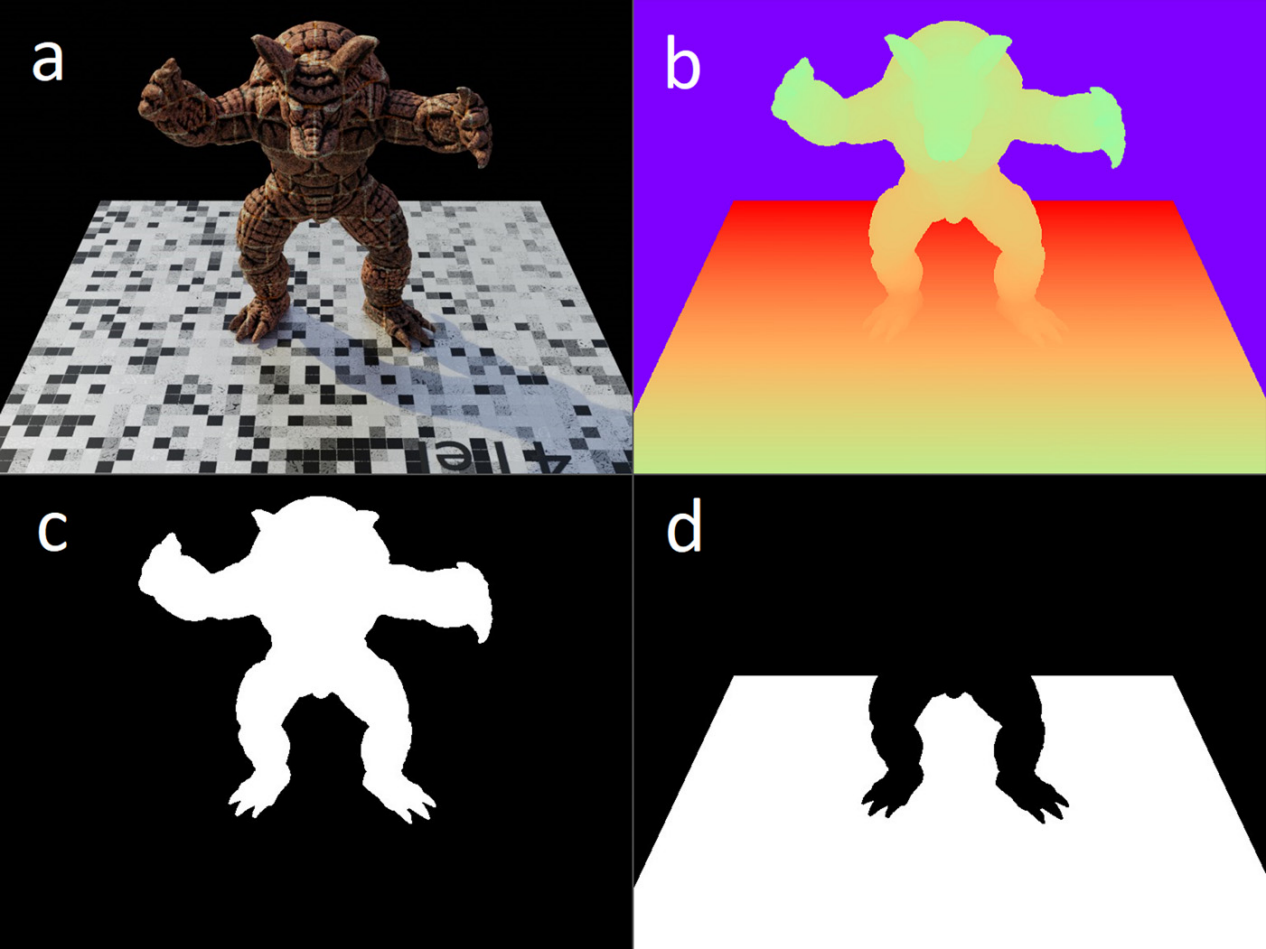
Illustration of the (a) image rendering, (b) ground truth depth map, (c) subject segmentation masque, (d) ground plane segmentation masque.

For the PASMVS dataset, all renderings are set to a fixed resolution of 768 × 576 pixels and exported as JPG image file format (Fig. 3a). The camera's sensor width is fixed at 36 mm with the focal length configured as either 35 mm or 50 mm. All images are rendered in perspective mode. The filename of every image is padded to a fixed length of eight characters, e.g. “00000000.jpg”.

## 3 cams

For every camera view stored in the “blended_images” folder, a corresponding camera information text file is provided. The filename of every camera file is padded to a fixed length of eight characters, e.g. “00000000_cam.txt”. The homogenous extrinsic (Euclidean rotation matrix alongside the translation vector) and intrinsic matrices are calculated from the “scene.csv” file, reducing the amount of post-processing required. For the last line of the camera file, the first and last terms refer to the minimum and maximum depth values of the geometry. The second and third terms refer to the step distance and number of depth hypotheses for a neural network implementation [1] respectively. The procedure for transforming the camera data from Blender's coordinate system to the intrinsic and extrinsic matrices provided in the text files, is detailed in the PASMVS data repository [11]. An example of the camera file content is provided:

extrinsic
−0.7069161 −0.7072961 0.0013672 −0.0105681
−0.3561577 0.3542949 −0.8646542 0.3330364
0.6110821 −0.6117249 −0.5023657 2.6840054
0.0000000 0.0000000 0.0000000 1.0000000
intrinsic
1066.6666667 0.0000000 384.0000000
0.0000000 1066.6666667 288.0000000
0.6634703 0.0175639 128.0 2.9116493

## 4 Masks

For every camera view stored in the “blended_images” folder, two binary masque images are generated. The masks match the resolution, filetype and naming scheme of the corresponding colour rendering. Camera rays intersecting the target geometry are set equal to one (white pixels), with the remaining pixels of the masque set equal to zero (black pixels). The masque for the model (Fig. 3c) and ground plane (Fig. 3d) are stored as “00000000obj.jpg” and “00000000gr.jpg” respectively, where the number again refers to the identification number of the camera view.

## 5 rendered_depth_maps

For every camera view stored in the “blended_images” folder, a corresponding ground truth depth map is provided (Fig. 3b). The depth maps match the resolution and naming scheme of the corresponding colour rendering, i.e. “00000000.pfm”. The rendered depth maps represent the distance measured from the camera's principal point to the intersecting scene geometry, for every pixel of the camera's imaging sensor. For empty space where there is no geometry, a distance value of zero is assigned. The depth map matrices, represented by float32 NumPy arrays, are serialised and stored in a PFM file format [1]. An example software implementation is provided [11] to read and write the PFM files.

The models, each including the ground plane, are exported to scale as a single stereolithography (STL) file. The compressed dataset archive is available from the online repository [11].

## 6 Experimental design, materials and methods

**Fig. 4:**
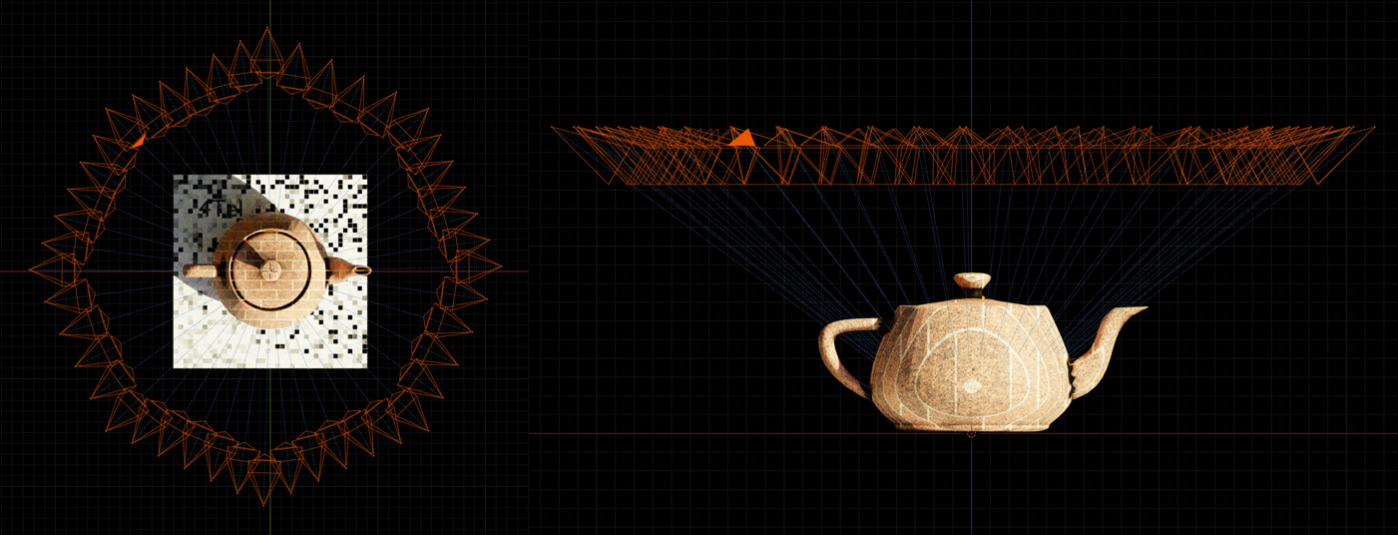
Positioning and orientation of the 45 cameras used for all the scenes.

Blender, the open source animation, graphics, and modelling software suite, was primarily used to create the dataset. Blender's implementation of the Cycles rendering engine provides the required fidelity and realism required for specular and diffuse material properties. Most of the materials can be classified as highly specular, with a smaller selection providing diffuse properties as a reference. The ground plane is unique for every scene, where the random seed for the checkboard colour and noise pattern is updated. The ground plane serves as additional feature points during the reconstruction process. If required, the ground plane can be removed using the appropriate binary segmentation masque. The 45 camera views per scene follow a circular path around the model (Fig. 4), with small perturbations added through noise modifiers to replicate more realistic deviations that would normally occur during image acquisition. The camera is rotated about the centre of the model, around the Z-axis. A constraint is added to the camera, automatically locking the camera view in the direction of the model. The camera position and orientation remain constant for all 400 scenes of the dataset.

The application of HDR environmental textures for lighting improves the realism considerably. The textures replicate the high-dynamic range of the illumination conditions encountered, avoiding the need to design the lighting environment manually. The orientation and rotation of the environmental textures were kept constant for all the scenes. Due to the specularity of the materials, the rendering engine was configured to use a larger sample size of 196, with all post-processing steps such as noise reduction disabled. Blender's internal Python API was used to fully automate the creation of the dataset, automating the cycling required for the environment textures, model visibility and camera focal length. The output file paths from Blender's compositor was also updated alongside automatic scene folder creation by the Python script.

For generating the ground truth depth maps, the camera's Z-buffer *distance* data is initially stored using the OpenEXR [17] file format. Due to the custom pinhole camera model implement by Blender, the Z-buffer of the camera provides the *distance* map, instead of the required *depth* map. This distortion is corrected [18] during post-processing in Python, using the known camera intrinsic properties, and with the corrected depth map exported in the serialized PFM file format. A fixed output resolution of 768 × 576 pixels is used for all the output files, namely colour rendering, binary segmentation masks and depth maps. A high-resolution (2048 × 1536 pixels) version of the dataset will be made available in the near future.

## CRediT authorship contribution statement

**André Broekman:** Conceptualization, Data curation, Formal analysis, Investigation, Methodology, Software, Validation, Visualization, Writing - original draft, Writing - review & editing. **Petrus Johannes Gräbe:** Funding acquisition, Project administration, Resources, Supervision, Writing - review & editing.

## Declaration of Competing Interest

The authors declare that they have no known competing financial interests or personal relationships which have, or could be perceived to have, influenced the work reported in this article.

## Appendix Supplementary materials

Image, application 1
